# Occupational health and safety characteristics of agricultural workers in Adana, Turkey: a cross-sectional study

**DOI:** 10.7717/peerj.4952

**Published:** 2018-06-01

**Authors:** Ramazan Azim Okyay, Ferdi Tanır, Pelin Mutlu Ağaoğlu

**Affiliations:** 1 Faculty of Medicine, Department of Public Health, Kahramanmaraş Sütçü İmam University, Kahramanmaraş, Turkey; 2 Faculty of Medicine, Department of Public Health, Çukurova University, Adana, Turkey

**Keywords:** Occupational health, Turkey, Agricultural workers

## Abstract

**Background:**

Among agricultural workers, especially in the seasonal migratory ones, housing and hygiene related issues, occupational accidents, low levels of education, poverty and absence of social security problems emerge as significant public health problems. This study aims to compare migrant-seasonal workers (MSWs) and resident agricultural workers (RAWs) in terms of socio-demographic characteristics and occupational health and safety in Adana, one of Turkey’s most important agricultural cities.

**Methods:**

This cross-sectional study was conducted on RAWs and MSWs, aged 15–65, operating in the province of Adana. The calculated sample sizes for both MSWs and RAWs were distributed using stratified simple random sampling to five districts of Adana.

**Results:**

The mean age of the 798 participating agricultural workers was 34.6 ± 14.2. Of the RAWs, 78.8% and of the MSWs 57.0% were male; 5.8% of RAWs and 32.8% of MSWs were illiterate. The mean number of people in the households of the participating workers was 5.1 for RAWs and 6.6 for MSWs. Of the RAWs, 20.5% were not covered by any social security scheme while this percentage was 35.1% in MSWs. RAWs worked 9.9 h a day while MSWs worked 10.9 h a day. Of the agricultural workers, 12.9% had injuries caused by occupational accidents.

**Discussion:**

Agricultural workers, who are a large part of Turkey’s economically active population, do not have healthy and safe working conditions. New regulations in the fields of social security, record keeping, monitoring, supervision, education and occupational health have been implemented recently to solve these problems. Despite the recent improvements there are still some problematic issues in the auditing of the necessary practices.

## Introduction

Agriculture is one of the most dangerous fields of work worldwide (Svendsen, Aas & Hilt, 2014). The causes of accidents and the poor health of workers in this sector are many, but often include: working with machines, vehicles, tools and animals, exposure to excessive noise and vibration, and others (Waggoner et al., 2013).

According to the International Labour Organization (2010), agriculture is the second greatest source of employment worldwide after the services sector. In addition, women and children work in agriculture, and seasonal migratory labor is widespread (Arcury & Quandt, 2007).

Agricultural workers are exposed to various risks due to the nature of agricultural production and these risks are more intense than other sectors. This is due to many agriculture-specific factors such as the dependence of agriculture on natural conditions, the importance of seasonality, the density of unpaid family labor, the low level of education and income instability. For this reason, the concept of work safety is becoming even more important for the agriculture sector and for those who work in this sector (Simsek, Doni & Koruk, 2012).

Turkish Statistical Institute (TURKSTAT) (2018) data indicates that the population employed in agriculture in Turkey is 19.4% of total employment in 2017.

Adana is the most remarkable city in Turkey’s Eastern Mediterranean Region in the Cukurova Delta, which is the second widest delta in the Mediterranean Basin just after the Nile Delta. Agriculture sector is an important source of employment in the city and migrant-seasonal workers (MSWs) also flock to the region especially in the spring and summer months. Therefore, this study aims to compare MSWs and resident agricultural workers (RAWs) in terms of socio-demographic characteristics and occupational health and safety in Adana, one of Turkey’s most important agricultural cities.

## Materials and Methods

### Study design

The study used the survey outcomes of a public health dissertation written in the 2013–2014 academic year in Cukurova University in Adana. This cross-sectional study presents the socio-demographic characteristics of agricultural workers and the outcomes regarding their occupational health and safety.

### Sampling

The population of the study included RAWs who were actively involved in agriculture, and whose ages ranged between 15 and 65, along with MSWs who worked in Adana temporarily.

The number of farmworkers in the region was not exactly known. Thus, the minimum sample size calculated according to an unknown universe size, in 95% confidence interval and assuming 5% margin of error, was 384 persons for RAWs using the Epi-Info program. A sample for MSWs was also specified for comparison.

Ceyhan, Seyhan, Yuregir, Karatas and Yumurtalik are the districts where agricultural activities are most intensively conducted in Adana. Therefore, we aimed to reach the agricultural workers in those five districts. Since the temporary settlement locations of MSWs were not fully known in advance, the regions where they can be found were determined among the regions visited by Adana Directorate of Provincial Food Agriculture and Livestock within the framework of strategy and action plan for a national project. Later, 28 temporary settlement locations for MSWs and 28 villages for RAWs were determined and the sample was distributed using stratified simple random sampling for each district.

### Data collection and measurement

A questionnaire querying the socio-demographic characteristics and occupational health and safety status developed by the researcher was applied to all participants. Since the questionnaire in the public health dissertation mentioned above was very lengthy, only the questions related to the data used in this article were extracted, translated and presented as a Supplemental File.

The data were collected between March 2013 and October 2013 in interviews by pollsters who had been trained beforehand. After the preliminary study conducted for the sake of the functionality of the survey, data were acquired from 798 agricultural workers, of whom 402 were MSWs, and 396 were RAWs, which is slightly more than the calculated sample size.

The statistical analysis was done using chi-square and *t* tests, assuming *p* < 0.05 to be statistically significant. The authors used the SPSS program (SPSS, Chicago, IL, USA) to do the statistical analysis.

### Ethical considerations

The research was completed in accordance with the Helsinki Declaration and the data were used provided that the confidentiality of all participants is preserved. Ethical approval was obtained from the Scientific Ethical Board of Cukurova University (Decision number 2012/10), and informed consent was obtained from the participants.

## Results

### The demographic characteristics of the agricultural workers

The mean age of the 798 participating agricultural workers was 34.6 ± 14.2. The mean age of the RAWs was 38.9 ± 13.6 and that of the MSWs was 30.5 ± 13.5. The distribution of population was younger for MSWs than for RAWs, and the difference was significant (*t* = 8.703, *p* < 0.0001). The mean number of people in the households of the participating workers was 5.1 ± 2.3 for RAWs and 6.6 ± 2.4 for MSWs. The average number of children in their households was 3.1 ± 2.1 for RAWs and 4.5 ± 2.8 for MSWs. The number of total persons (*t* = 8.561, *p* < 0.0001) and children in the households (*t* = 6.634, *p* < 0.0001) were significantly higher for MSWs than for RAWs (Table 1).

**Table 1:** Demographic data on age and number of household members of agricultural workers.

Socio-demographic characteristics	RAWs (number = 396)		MSWs (number = 402)		*p*^a^
	Mean ± SD	Med (min–max)	Mean ± SD	Med (min–max)	
Age (years)	38.9 ± 13.6	39(15–65)	30.5 ± 13.5	26(15–65)	<0.0001
Mean number of household members	5.1 ± 2.3	5(1–15)	6.6 ± 2.4	6(1–13)	<0.0001
Mean number of children in households	3.1 ± 2.1	3(0–18)	4.5 ± 2.8	4(0–16)	<0.0001

**Notes:**

a Student’s *t*-test.

α, 0.05.

Of the RAWs, 78.8% and of the MSWs 57.0% were male (χ^2^ = 43.512, *p* < 0.0001). The proportion of illiteracy was found 5.8% in RAWs and 32.8% in MSWs. In general RAWs had a better educational levels (χ^2^ = 134.959, *p* < 0.0001). Of the RAWs, 74.0% and of the MSWs 60.4% were married. Consanguineous marriage was found to be significantly higher in MSWs (χ^2^ = 28.095, *p* < 0.0001). Of the RAWs, 20.5% were not covered by any social security scheme while this was 35.1% in MSWs (χ^2^ = 21.235, *p* < 0.0001). The demographic data of the agricultural workers, except for their ages and household members, are presented in Table 2.

**Table 2:** The distribution of demographic data of agricultural workers by groups.

Socio-demographic characteristics	RAWs (number = 396)		MSWs (number = 402)		Total		*p*^b^
	Number	%^a^	Number	%^a^	Number	%^a^	
**Gender**							
Male	312	78.8	229	57.0	541	67.8	0.0001
Female	84	21.2	173	43.0	257	32.2	
**Educational level**							
Illiterate	23	5.8	132	32.8	155	19.4	0.0001
Literate	24	6.1	45	11.2	69	8.6	
Elementary	273	68.9	211	52.5	484	60.7	
High school	66	16.7	14	3.5	80	10.0	
Higher education	10	2.5	0	0.0	10	1.3	
**Marital status**							
Married	293	74.0	243	60.4	536	67.2	0.0001
Single	100	25.3	156	38.8	256	32.1	
Widow/widower/divorced	3	0.8	3	0.7	6	0.7	
**Consanguineous marriage**							
No	253	85.5	162	66.1	415	76.7	0.0001
Yes	43	14.5	83	33.9	126	23.3	
**Degree of consanguineous marriage**							
First cousin	35	81.4	70	84.3	105	83.3	0.802
Second cousin	8	18.6	13	15.7	21	16.7	
**Having social security**							
No	81	20.5	141	35.1	222	27.8	0.0001
Yes	315	79.5	261	64.9	576	72.2	

**Notes:**

a Column percentage.

b Pearson chi-square test.

α, 0.05.

### The occupational characteristics of the agricultural workers

The participants reported that they worked 10.4 ± 2.2 h a day. RAWs worked 9.9 ± 2.6 h a day while MSWs worked 10.9 ± 1.7 h a day, (*t* = 6.222, *p* < 0.0001); RAWs worked 6.2 ± 1.4 days a week and MSWs worked 6.4 ± 1.1 days a week (*t* = 2.437, *p* = 0.015). It was found that MSWs worked significantly longer than the RAWs.

Of the agricultural workers, 12.9% (103) had injuries caused by occupational accidents. This was 16.9% (67) in RAWs and 9% (36) in MSWs. It was found that the RAWs had more accidents than the MSWs (χ^2^ = 11.255, *p* = 0.001). The distribution of occupational accidents and injuries among agricultural workers is presented in Table 3.

**Table 3:** Types of occupational accidents and injuries among agricultural workers.

Location and type of the injury	RAWs	MSWs	Total	*p*^b^
	%^a^	%^a^	%^a^	
Upper extremity injury	32.8	44.4	36.9	
Lower extremity injury	31.3	30.6	31.1	
Head and neck injuries	4.5	8.3	5.8	
Chest, abdominal and back injuries	10.5	0.0	6.8	0.349
Upper and lower extremity injury	11.9	11.1	11.6	
Poisoning	9.0	5.6	7.8	
**Total**	100	100	100	

**Notes:**

a Column percentage.

b Pearson chi-square test.

α, 0.05.

Of the participants, 83.2% used at least one personal protective equipment (PPE). It was found that hats were the most commonly used PPE among the participants. The distribution of the PPEs used by the workers is shown in Table 4.

**Table 4:** The distribution of the PPEs used by agricultural workers.

PPE type	RAWs		MSWs		Total		*p*^b^
	Number	%^a^	Number	%^a^	Number	%^a^	
**Work clothes**							
Yes	174	43.9	138	34.3	312	39.1	0.006
No	222	56.1	264	65.7	486	60.9	
**Hat**							
Yes	319	80.6	316	78.6	635	79.6	0.539
No	77	19.4	86	21.4	163	20.4	
**Gloves**							
Yes	185	46.7	172	42.8	357	44.7	0.286
No	211	53.3	230	57.2	441	55.3	
**Mask**							
Yes	43	10.9	14	3.5	57	7.1	0.0001
No	353	89.1	388	96.5	741	92.9	
**Goggles**							
Yes	42	10.6	3	0.7	45	5.6	0.0001
No	354	89.4	399	99.3	753	94.4	

**Notes:**

a Column percentage.

b Pearson chi-square test.

α, 0.05.

It was found that 72.9% of RAWs and 26.6% of MSWs did pest control and 73.5% of RAWs and 28.4% of MSWs were present at the field during pest control and the differences between the groups were significant (χ^2^ = 171.531, *p* < 0.0001); (χ^2^ = 162.533, *p* < 0.0001). Appropriate occupational safety behaviors of agricultural workers were not at the desired levels. For example, 8.1% of RAWs and only 0.9% of MSWs were wearing overalls. Occupational safety behaviors of the agricultural workers who did pest control or were present at the field during pest control (exposed to pesticides) is shown on Table 5.

**Table 5:** The occupational safety behaviors of agricultural workers who were exposed to pesticides.

Occupational safety behaviors related to pest control	RAWs	MSWs	Total	*p*^b^
	%^a^	%^a^	%^a^	
**Using mask**				
Yes	22.4	12.9	19.7	0.038
No	77.6	87.1	80.3	
**Wearing gloves**				
Yes	26.1	28.4	26.8	0.629
No	73.9	71.6	73.2	
**Covering the hair/wearing a bonnet**				
Yes	13.6	12.1	13.1	0.748
No	86.4	87.9	86.9	
**Wearing overalls**				
Yes	8.1	0.9	6.1	0.005
No	91.9	99.1	93.9	
**Washing the hands and face after pest control**				
Yes	61.7	58.6	60.8	0.576
No	38.3	41.4	39.2	
**Having a shower after pest control**				
Yes	48.1	44.8	47.2	0.584
No	51.9	55.2	52.8	
**Washing pest control clothes separately**				
Yes	24.4	10.3	20.4	0.001
No	75.6	89.7	79.6	

**Notes:**

a Column percentage.

b Pearson chi-square test.

α, 0.05.

## Discussion

The average age of the participating agricultural workers was 34.6. However, MSWs were younger than RAWs. The participants’ mean number of household members and mean number of children were found to be above the national averages (Turkish Statistical Institute (TURKSTAT), 2016a, 2016b). The participants in general and MSWs in particular had crowded households with many children, which may indicate the insufficiencies of the families in education, health and income.

Although the educational levels of RAWs in this study were relatively higher than MSWs, the literacy percentage and educational levels of agricultural workers were still low in general. A study by Kutlu (2011) found that 60% of the MSWs did not attend school, while a study by Yavuz (2013) found that 32% of them were illiterate. These findings suggest that agricultural workers, particularly the MSWs, have low education levels in Turkey.

We found a high proportion of consanguineous marriage in this study. A majority of these marriages were among first cousins, which were most risky. The rate of consanguinity was reported be approximately 20–25% in Turkey (Tunçbilek & Özgüç, 2007). We believe that educational interventions about the negative health consequences of consanguineous marriages are necessary in agricultural workers.

Of RAWs in this study, 20.5% were not covered by any social security scheme while this was 35.1% in the MSWs. According to the Food and Agriculture Organization, less than 20% of the agricultural workers worldwide are covered under a social security scheme that covers all kinds of health services (Powell et al., 2006). A study from the United States reported that only 23% of agricultural workers had some kind of health insurance (Carroll et al., 2005). While these results indicate that the social security status of agricultural workers in Turkey is better than that of workers in many countries, substantial amount of workers are still not covered under any social security scheme.

The participants in this study worked for more than 10 h a day. It is known that extended working hours are associated with stress, fatigue and chronic conditions such as cardiovascular and musculoskeletal disorders (Johnson & Lipscomb, 2006). Arrangements should be made to prevent agricultural workers from excessive working in order avoid negative health outcomes.

Of the participants in this study, 12.9% reported having had occupational injuries. Another study conducted in Australia found this rate to be as high as 5.6% (Safe Work Australia, 2013). We found that vast majority of injuries were extremity injuries. Similarly a study by Xiang et al. (2000) conducted in China reported a 68.5% of extremity injuries, which is consistent with the findings of this study. This shows that in countries where manual methods are still in use, injuries to the extremities are widespread and are caused by the equipment used by the workers or by heavy loads.

Hats and gloves were, respectively, the most frequently used PPEs for the participants of this study. There was no difference between the RAWs and the MSWs in terms of the use of these materials, while the use of work clothes, masks and goggles among the RAWs was significantly higher. In their study, Yavuz (2013) argued that 78.8% of the workers did not use any equipment against the dust and 96% did not use earplugs. These findings are consistent with this study’s results and show the insufficient use of PPEs by the workers.

This study had shown that 73.0% of RAWs and 26.6% of MSWs did pest control. An analysis of the occupational health and safety behaviors of agricultural workers who did pest control or were present at the field during pest control (exposed to pesticides) revealed that both RAWs and MSWs demonstrated inappropriate and unsafe attitudes. Our findings are in accordance with other studies carried out by Yavuz (2013), Ergönen (2000) and Sahin et al. (2010). Unfortunately, agricultural workers who do pest control in Turkey, do not adequately use PPEs and their hygienic behaviors are far from the desired levels.

Our study demonstrated the long-standing problems of agricultural workers. Previous research mostly focused on either RAWs or MSWs. However in this study, both RAWs and MSWs were included, which enabled us to make comparisons between groups. About 28 temporary settlement locations and 28 villages chosen from five districts with the most intense agricultural activity were visited for data collection. Thus, our study is quite capable of representing the population. On the other hand, there are some limitations to our study. Since the data was collected using questionnaires, the accuracy of the participants’ responses was not clear. Additionally memory factors might affect the responses. Further, due to restraints of time and resources, we had to make sampling and we could not visit all districts.

## Conclusion

Agricultural workers, who are a large part of Turkey’s economically active population, do not have healthy and safe working conditions. This study demonstrated that a considerable portion of these workers were not covered under any social security scheme, and a majority of them were working for long hours. They tended to have injuries to their extremities during their work. Workers’ attitudes and behaviors regarding occupational health and safety were not plausible. New regulations in the fields of social security, record keeping, monitoring, supervision, education and occupational health have been implemented recently to solve these problems. Despite the recent improvements, there are still problematic issues in the auditing of the necessary practices. Further studies focusing on the deficiencies in supervision and auditing should be carried out in collaboration with all stakeholders such as agricultural workers, occupational safety and health professionals, local authorities and the government. We hope that the epidemiologic data provided in this study may shed light on future studies regarding occupational health and safety in agricultural sector and help policy makers in this regard.

## Supplemental Information

10.7717/peerj.4952/supp-1 Supplemental Information 1 Raw data.

10.7717/peerj.4952/supp-2 Supplemental Information 2 Survey form.

## Additional Information and Declarations

### Data Availability

The following information was supplied regarding data availability: The raw data are provided in a Supplemental File.
