# Supplementary material for: Occupational health and safety characteristics of agricultural workers in Adana, Turkey: a cross-sectional study
Source: PeerJ. 2018 Jun 1;6:e4952. doi: 10.7717/peerj.4952 (PMC5985757; doi:10.7717/peerj.4952)
Supplement: Supplemental Information 2 [file peerj-06-4952-s002.docx]

**AGRICULTURAL WORKERS SURVEY FORM**

**Socio-demographic Characteristics**

**1. Which group (RAWs or MSWs:**

**2. Total number of people living in the household:…………………................................**

**3. Name:……………………………………………………………………………………..**

**4. Age: ………..**

**5. Gender:** 1.Male 2.Female

**6. Educational status**

1. Illiterate 2.Literate 3.Primary school

4.Middle school 5.High school 6.College

**7. Marital status**

1. Married 2. Single 3.Divorcee/widow

**8. Is there a consanguineous marriage?**

1. No 2.Yes (Please specify)………………...................................

**9. How many children do you have? ……………….**

**10. Are you covered under any social security scheme?**

1. No 2. Yes......................................................................................

**Occupational Status**

**11. Have you ever performed spraying pesticides on the field?**

1.Yes

2.No

**12. Have you ever been present on the field during spraying process?**

1.Yes

2.No

**13. Which of the following actions do you do before/during or after the process of spraying?**

1. Using mask

2. Wearing gloves

3. Covering the hair/Wearing a bonnet

4. Wearing overalls

5. Washing the hands and face after pest control

6. Having a shower after pest control

7. Washing pest control clothes separately

**14. Have you ever been injured during work?**

1. No

2. Yes (Please specify) …………………………………………………………….............

**15. Do you use personal protective equipment while working?**

1.Yes

2.No

**16. Which of the following do you use while working?**

1.Work Clothes 2.Hat 3. Gloves

4.Mask 5. Goggles

**17. How many hours do you work per day? ......................**

**18. How many days do you work per week?......................**
